# Epidemiological Changes in Respiratory Viral Infections in Children: The Influence of the COVID-19 Pandemic

**DOI:** 10.3390/v15091880

**Published:** 2023-09-05

**Authors:** Teresa Almeida, João Tiago Guimarães, Sandra Rebelo

**Affiliations:** 1 Department of Clinical Pathology, São João Hospital University Center, 4200-319 Porto, Portugal; tiago.guimaraes@chsj.min-saude.pt (J.T.G.); srebelo1@gmail.com (S.R.); 2 Department of Biomedicine, Faculty of Medicine, University of Porto, 4200-319 Porto, Portugal; 3 EPIUnit—Institute of Public Health, University of Porto, 4050-600 Porto, Portugal; 4 Laboratory for Integrative and Translational Research in Population Health (ITR), 4050-600 Porto, Portugal; 5 Institute for Research and Innovation in Health (i3S Consortium), University of Porto, 4200-135 Porto, Portugal

**Keywords:** respiratory virus, children, pandemic, acute respiratory infection, COVID-19

## Abstract

Background: Viruses are the major cause of acute respiratory infections in children, causing important morbimortality. Before the COVID-19 pandemic, in temperate regions, respiratory viruses displayed a typical seasonality in transmission. A disruption in this pattern was observed in several countries during the pandemic, with low prevalence during the typical season, and an interseasonal rise. We evaluated the effects of the COVID-19 pandemic in the epidemiology of non-COVID viral respiratory infections in children, in a tertiary care hospital in Portugal. Methods: Between March 2020 and August 2022, nasopharyngeal samples from children with respiratory symptoms in the Emergency Department (ED) and the Pediatric Ward were tested for RSV, influenza and other respiratory viruses, by real-time reverse transcriptase PCR (RT-PCR). Results: A seasonal variation was observed from 2018 to 2020, with prevalence increasing in winter (mainly RSV and influenza). In the winter of 2020/21, when measures to mitigate SARS-CoV-2 transmission were stricter, there was a disruption of the seasonal pattern, with unusually low numbers. In the summer of 2021, when measures were being relaxed, there was an atypical rise. In June 2021, RSV was first detected and peaked in October. Influenza (Influenza A H3) was detected for the first time in February 2022, peaking in March/April. Conclusions: These findings show a disruption of the seasonality of viral respiratory infections in children during the pandemic, with a virtual elimination during the months of usually higher prevalence, and a subsequent out-of-season increase, coinciding with variations in the measures implemented to control the SARS-CoV-2 transmission, and confirming their efficacy.

## 1. Introduction

Acute respiratory infections are the most frequent infectious diseases in children. They can occur between six and eight times per year, constitute one of the major causes of hospitalization, particularly under two years old, and represent a huge burden of lost working days for parents or caregivers [1]. The vast majority of these infections have a viral etiology, and the agents most often responsible in pediatric age are the two types of respiratory syncytial virus (RSV A-B), rhinoviruses (RVs), the four types of parainfluenza virus (PIV 1–4), influenza viruses A-B and adenoviruses [2]. Other agents include the human metapneumovirus (hMPV), the human bocavirus (hBoV) and the human coronaviruses (CoV). In 2019, a new coronavirus (SARS-CoV-2) emerged in China, causing coronavirus disease 2019 (COVID-19), leading to a global pandemic and a public health emergency declared by the World Health Organization (WHO) [3].

Prior to the beginning of the COVID-19 pandemic, most respiratory viruses showed a typical seasonal oscillation of their outbreaks in temperate regions, with influenza, CoV, and RSV infections peaking in the winter months; adenovirus, hBoV, hMPV and RVs detected throughout the year; and some enteroviruses increasing during the summer [4,5]. However, since the onset of the COVID-19 pandemic, reports from the US, Australia and several European countries showed a change in the epidemiology of respiratory viruses, with low numbers during the typical season and an interseasonal outbreak [6,7,8,9,10]. Possible explanations for this reduced occurrence are the implementation of non-pharmacological interventions to prevent the transmission of SARS-CoV-2, including mandatory face mask use, lockdowns, school closures, travel restrictions and other public health and physical distancing measures, and the viral competition between SARS-CoV-2 and other respiratory viruses [11,12,13,14]. We provide a description of a new trend in children’s respiratory infections epidemiology, from March 2020 to August 2022, using data collected in a tertiary care University Hospital in Northern Portugal.

## 2. Materials and Methods

### 2.1. Specimen Selection

Between March 2020 and August 2022, nasopharyngeal swabs collected in VTM (viral transport medium) for SARS-CoV-2 testing in children (aged 0 to 18 years) from the Emergency Department (ED) as well as in children admitted to the Pediatric Ward with respiratory symptoms were randomly selected for real-time reverse transcriptase PCR (RT-PCR) testing for RSV, influenza and other respiratory viruses.

### 2.2. Specimen Testing

RT-PCR analysis was performed on the GeneXpert^®^ system with Xpert^®^ Xpress Flu/RSV (Cepheid Inc., Sunnyvale, CA, USA; subsequently referred to as the Xpert Flu/RSV assay) [15,16], on the Cobas^®^ 6800 system with Cobas^®^ Influenza A/B & RSV UC Test (Roche Molecular Systems, Pleasanton, CA, USA; subsequently referred to as the Cobas Influenza/RSV assay) [17], on the BioFire^®^ FilmArray^®^ 2.0 system with Biofire^®^ Respiratory Panel 2.1 (BioFire Diagnostics, Salt Lake City, UT, USA; subsequently referred to as the Biofire Respiratory Panel) [18], and using Allplex^®^ Respiratory Panel Assays (Seegene, CA, USA; subsequently referred to as the Allplex respiratory panel) [19]. These assays were performed according to the manufacturer’s instructions.

### 2.3. Data Collection

Data from clinical records were retrospectively collected by the authors from a period between January 2018 and August 2022, using the Laboratory Information System (Clinidata^®^ XXI and Clinidata^®^ Web—Maxdata, Portugal) and SClínico^®^ v 2.7 (patient record software). Information collected included: sex, age, medical history and medication, previous hospital admissions and surgeries, vaccination status, epidemiological context, symptoms and duration, physical examination, SARS-CoV-2 diagnosis, treatment (including antibiotic prescription), hospital admission and duration, and destiny after discharge.

### 2.4. Data Analysis

Data analysis was performed using Microsoft^®^ Excel for Mac, version 16.16.27.

### 2.5. Ethical Statement

No ethical consent was required as only aggregated and anonymized data were processed.

## 3. Results

In March and April 2021, when the non-pharmacological measures to prevent SARS-CoV-2 transmission were stricter in Portugal, during a national state of emergency, samples that were collected for SARS-CoV-2 testing were selected and tested for RSV and influenza (Xpert Flu/RSV assay or Cobas Influenza/RSV assay). From the 116 samples that were tested with the Xpert Flu/RSV assay, from children originating from the Emergency Department (ED), including 56 girls (48%) and 60 boys (52%), with ages between one week and 18 years (mean 5.4 ± SD 5.5), none were positive for RSV or influenza. The 2132 samples that were tested with the Cobas Influenza/RSV assay, including children and adults from the ED, were all negative for RSV and influenza.

**Figure 1:**
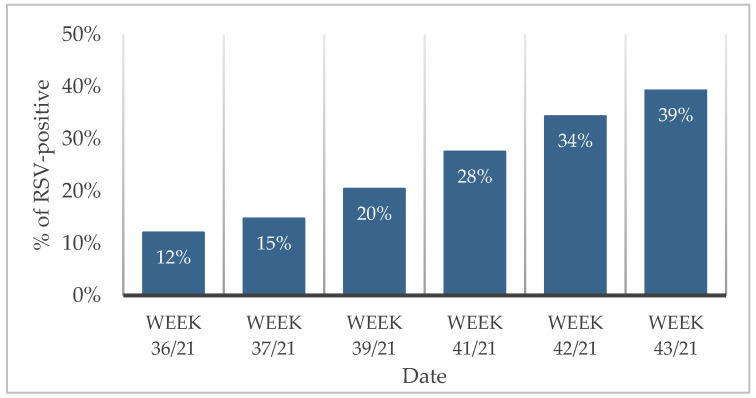
Percentage of RSV-positive children from the ED in different weeks (week 36/2–5 September 2021, week 37/8–12 September 2021, week 39/28–29 September 2021; week 41/12–13 October 2021; week 42/20–21 October 2021, week 43/27 October 2021).

In late July 2021, after one and a half years without an expression of respiratory virus in children other than SARS-CoV-2, we detected the first RSV infections in children admitted to the Pediatric Ward. This occurred in a phase when the non-pharmacological measures were being relaxed in Portugal, due to control and low numbers of SARS-CoV-2 infections. In September and October 2021, 626 children from the Emergency Department with respiratory symptoms that provided a nasopharyngeal swab for SARS-CoV-2 testing, were also tested with the Xpert Flu/RSV assay. Of these, 293 (46.8%) were female and 333 (53.2%) were male, and the ages ranged from 5 days to 18 years (mean 4.2 ± SD 4.4). Of these children, 11 (2%) had tested positive for SARS-CoV-2. Overall, 141 (22%) tested positive for RSV and none for influenza. No co-infection of RSV and SARS-CoV-2 was detected. Comparing the RSV results from different consecutive weeks, a gradual increase in RSV positivity was evident: 14% (39/280), 20% (19/93), 28% (27/98), 34% (34/99) and 39% (22/96) (Figure 1).

**Figure 2:**
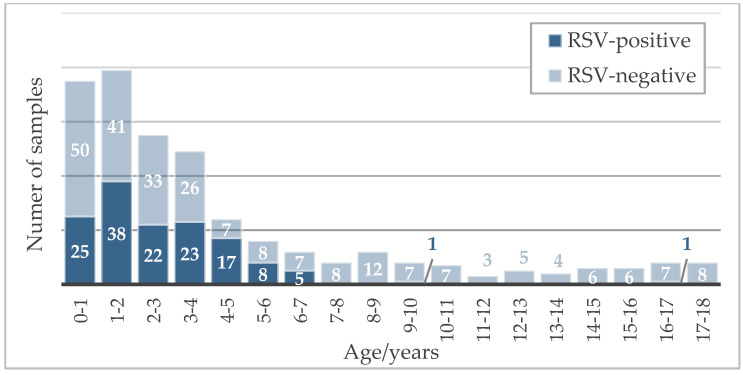
Age distribution of children tested for RSV infection from September to October 2021.

The 141 children that tested positive for RSV comprised 64 girls (45%) and 77 boys (55%), with ages ranging from 2 months to 16 years (medium 2.4 ± 2.0 SD). Figure 2 represents the tested children and RSV-positive cases by age.

The four most common diagnoses in the children testing positive for RSV were: common cold—32% (*n* = 45); non-specified viral infection—15% (*n* = 21); acute bronchiolitis—9% (*n* = 12); and otitis media—6% (*n* = 9). Of these patients, 15% (*n* = 21) returned to the ED for further treatment, and 2% (*n* = 3) required inpatient care. Antibiotic therapy was prescribed in 21% (*n* = 29) of the children, mainly due to a diagnosis of otitis media (*n* = 13), pneumonia (*n* = 7) or urinary tract infection (*n* = 4).

**Figure 3:**
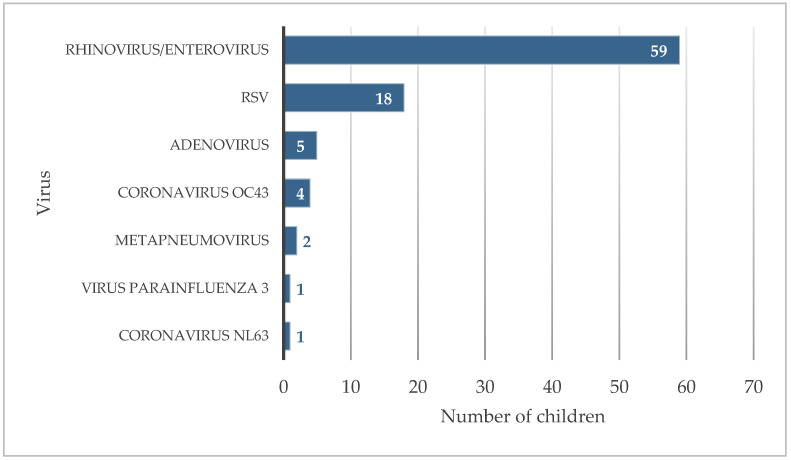
Respiratory viruses detected in children from the ED.

To better characterize all the agents, beyond RSV, causing these respiratory infections, nasopharyngeal samples from children in the ED throughout a 24 h interval (28 to 29 of September) were tested with the Biofire Respiratory Panel, an expanded multiplex PCR system with a syndromic approach that detects and identifies the pathogens most commonly associated with respiratory infections. Of the 86 children tested, 59 samples were positive for rhinovirus/enterovirus (69%), 18 for RSV (21%), 5 for adenovirus (6%), 4 for coronavirus OC43 (5%), 2 for metapneumovirus (2%), 1 for parainfluenza virus 3 (1%) and 1 for coronavirus NL63 (1%) (Figure 3). No agent was detected for 19 children (22%) and none of the children were positive for SARS-CoV-2. Of these children, 20 (23%) had a co-infection with two or more agents, the most frequent being co-infection with rhinovirus/enterovirus and RSV (11 children, 13%). Subsequent testing of the samples positive for rhinovirus/enterovirus using the Allplex respiratory panel showed that they were all rhinovirus-positive.

**Figure 4:**
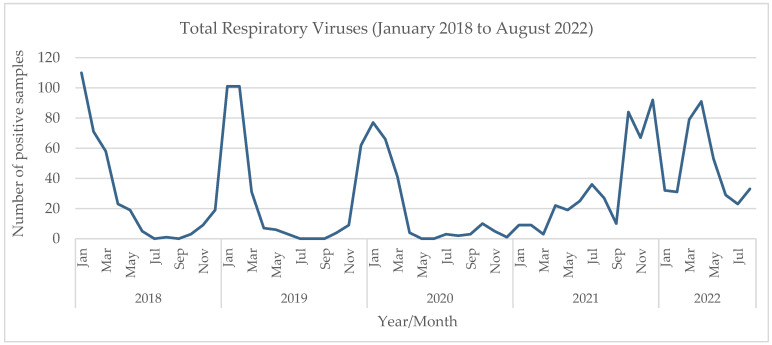
Detection of viruses in respiratory samples in children, January 2018 to August 2022.

**Figure 5:**
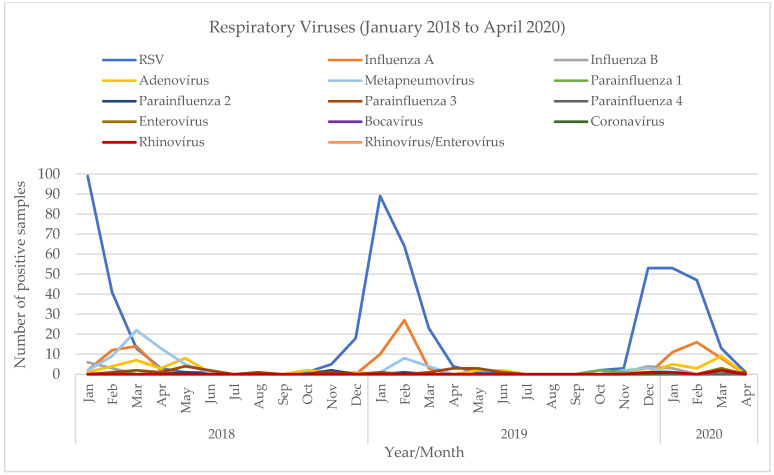
Detection of viruses in respiratory samples in children, January 2018 to April 2020.

**Figure 6:**
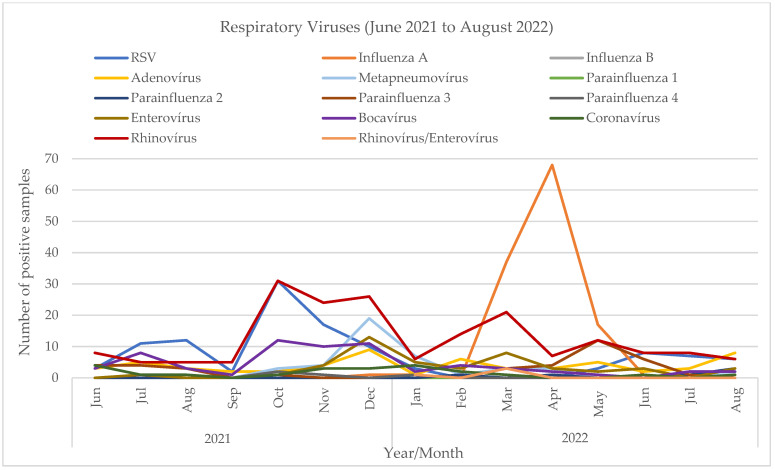
Detection of viruses in respiratory samples in children, June 2021 to August 2022.

Additionally, we performed a retrospective comparative analysis of all viruses detected in respiratory specimens from children in our hospital between January 2018 and August 2022. The results of that extended analysis show two different patterns (Figure 4). From 2018 to the winter of 2019/20 (Figure 5), before the beginning of the SARS-CoV-2 pandemic, there was a seasonal variation, with these viruses peaking in the winter, from November to March (the maximum number of total virus infections was 110 in January 2018, 101 in January and February 2019, and 77 in January 2020). The most prevalent viruses were RSV and influenza. In the winter of 2020/21, the first winter during the pandemic, when measures to mitigate SARS-CoV-2 transmission were stricter, there was a clear change in these viruses’ epidemiology. An unusually small number of infections were detected during the typical winter season (a maximum of 9 in January 2021)—mostly rhinovirus, and no RSV or influenza. Later, in the summer of 2021 (Figure 6), a period in which those measures were being relaxed, there was an atypical rise in viral infections (88 total infections between June and August 2021 vs. 6, 3 and 5 cases in 2018, 2019 and 2020, respectively). In July 2021, the first RSV infections were detected, and prevalence increased gradually, peaking in October with 31 cases. There were no influenza infections until January 2022, when Influenza A (H3) was first detected, and peaked afterwards in April with 68 cases. Although the weather was dry and the atmospheric temperature was higher than usual, there were 125 cases between March and May.

## 4. Discussion

Before the COVID-19 pandemic, in the Northern Hemisphere, there was a seasonal pattern of respiratory infections in children, with most influenza, CoV, and RSV infections occurring during the winter season from November to March, hMPV and RVs throughout the year, and some enteroviruses during the summer [4].

In Portugal, the first patient with a COVID-19 infection was diagnosed on 2 March 2020, in Porto. To mitigate the transmission of SAR-CoV-2, as in other countries, several public health measures were implemented, including lockdown, isolation of contacts, mandatory use of respiratory masks, social distancing, and school closings, among others. The adoption of these restrictive measures could potentially interfere with the epidemiology of other respiratory viruses, that have the same routes of transmission, as it was observed in other countries. In Australia, Foley et al. [7] reported a RSV surge during the spring and summer months, as physical distancing restrictions were relaxed, instead of the typical fall and winter months. Agha and Avner [6] showed a seasonal shift and delayed surge of RSV infections in young children in a New York City hospital. In Europe, van Summeren et al. [8] showed that RSV circulation stopped immediately after non-pharmacological interventions were implemented to control SARS-CoV-2 transmission in February/March 2020, and RSV epidemics started with several weeks delay from the usual winter, in 2021. This pattern is repeated in several publications in the literature [9,10]. This motivated the authors of this study to assess the impact of the pandemic on the prevalence of these infections in children.

Since the COVID-19 pandemic started, there was a decrease in non-SARS-CoV-2 viral infections. Indeed, the results show that in March and April 2021, during a phase of strict non-pharmacological interventions, no RSV or influenza infections were detected in our center. However, in the summer of 2021, a typically low-prevalence season, when SARS-CoV-2 incidence was lower and non-pharmacological measures were being relaxed in Portugal, RSV infections were detected for the first time in July. In September and October 2021, the number of RSV infections was unusually high and increasing progressively (Figure 1) This break in the seasonal pattern of respiratory infections and delayed surge is consistent with the results published in other countries.

The exhaustive investigation of the agents responsible for respiratory infections in children, using a syndromic approach with the application of a multiplex panel of respiratory pathogens, allowed us to observe that, in addition to RSV, there was also an uncharacteristic increase in infections with other respiratory viruses, such as rhinovirus, adenovirus or other coronaviruses (Figure 3). It is important to note that this screening for other respiratory viruses was carried out as never before, due to the tight surveillance that was in place at the time to prevent SARS-CoV-2 outbreaks. Consequently, the resulting data was completely new and difficult to interpret compared to previous years, in which these respiratory infections were clinically diagnosed and empirically treated without identifying the responsible agent.

The retrospective analysis of all viruses detected in respiratory samples from children in our hospital, from January 2018 to August 2022, allowed us to further visualize and document that break in seasonality. It showed that, prior to the beginning of the SARS-CoV-2 pandemic, a seasonal pattern was observed, with peaks of RSV and influenza in the winter months. That pattern was interrupted in the first winter of the pandemic (2020/21), with virtually no cases of the usually prevalent viruses in that season, coinciding with the increase in mitigation measures to prevent SARS-CoV-2 transmission. Then, in the summer of 2021, unlike previous years when the viral activity was very low, there was an increase in the numbers of respiratory viruses. RSV was first detected in an atypical month, June, and increased until October, when it reached a maximum. Influenza was not detected from March 2020, and reappeared only in January 2022, reaching a maximum in April and May 2022. A limitation of our study is the lack of a complete statistical analysis. However, the nature of the available dataset precluded us from executing such an evaluation with statistical quality, permitting only a comprehensive descriptive analysis of the assessed time frame. We believe that, given the primary aims of the article, this analysis ultimately constitutes a valuable contribution.

In summary, we describe the casuistic of viral respiratory infections in children in a tertiary care hospital in Portugal, in a timeline that comprises three different phases:

- Pre-pandemic: seasonality as described in the state of the art.
- Post-pandemic:
  - Initial pandemic phase: disappearance of all non-SARS-CoV-2 respiratory viruses;
  - Evolution: detection of the first RSV cases in July 2021, reintroduction of non-SARS-CoV-2 viruses, starting with out-of-season outbreaks, and probable evolution towards the restauration of the usual epidemiological patterns.

To our knowledge, this paper describes for the first time the epidemiological characterization of this type of infections in Portugal during this period.

In conclusion, we describe a change in the epidemiology of respiratory viral infections in Northern Portugal, with a virtual elimination of cases detected during the period of greater restrictions due to the COVID-19 pandemic, followed by an anticipated increase in cases in the summer, an unusual period, associated with the relaxation of restrictions due to greater control of the infection by SARS-CoV-2. Additionally, our findings corroborate the efficacy of the measures implemented to control the SARS-CoV-2 transmission.
